# Supplementary material for: The structural basis of divalent cation block in a tetrameric prokaryotic sodium channel
Source: Nat Commun. 2023 Jul 15;14:4236. doi: 10.1038/s41467-023-39987-0 (PMC10349818; doi:10.1038/s41467-023-39987-0)
Supplement: Supplementary file 1 — Supplementary Information [file 41467_2023_39987_MOESM1_ESM.pdf]

Supplementary Information

The structural basis of divalent cation block in a tetrameric prokaryotic sodium channel

Katsumasa Irie[1,2*], Yoshinori Oda[3], Takashi Sumikama[4,5], Atsunori Oshima[2,3,6,7], and Yoshinori Fujiyoshi[8,9].

[1]Department of Biophysical Chemistry, School of Pharmaceutical Sciences, Wakayama Medical University, 25-1, Shichibancho, Wakayama, 640-8156, Japan

[2]Cellular and Structural Physiology Institute (CeSPI), Nagoya University, Furo-cho, Chikusa, Nagoya, 464-8601, Japan.

[3] Department of Basic Medicinal Sciences, Graduate School of Pharmaceutical Sciences, Nagoya University, Furo-cho, Chikusa, Nagoya, 464-8601, Japan.

[4] PRESTO, JST, Kawaguchi, 332-0012, Japan

[5] Nano Life Science Institute (WPI-NanoLSI), Kanazawa University, Kanazawa, 920-1192, Japan

[6] Institute for Glyco-core Research (iGCORE), Nagoya University, Furo-cho, Chikusa-ku, Nagoya 464-8601, Japan

[7] Center for One Medicine Innovative Translational Research, Gifu University Institute

for Advanced Study, Gifu 501-11193, Japan.

[8] Cellular and Structural Physiology Laboratory (CeSPL), Advanced Research Institute, Tokyo Medical and Dental University, 1-5-45, Yushima, Bunkyo, Tokyo 113-8510, Japan

[9] CeSPIA Inc., 2-1-1, Otemachi, Chiyoda, Tokyo, 100-0004, Japan

*Address correspondence to: Katsumasa Irie, Department of Biophysical Chemistry, School of Pharmaceutical Sciences, Wakayama Medical University, 25-1, Shichibancho, Wakayama, 640-8156, Japan

Tel.: +81-73-488-1837; Fax: +81-73-488-1946; E-mail: kirie@wakayama-med.ac.jp

Supplementary Table 1. Data collection and refinement statistics

	**N49K_ Na**	**N49K_ Ca**	**L176Q^{NK} _Na**	**L176Q^{NK} _Ca**	**L176G^{NK} _Na**	**L176G^{NK} _Ca**
**PDB entry**	8H9O	8H9W	8H9X	8H9Y	8HA1	8HA2
**Wavelength (Å)**	1.00					
**Resolution range(Å)**	29.2-3.3 (3.4-3.3)	29.8-2.7 (2.8-2.7)	28.8-3.4 (3.5-3.4)	29.3-3.4 (3.5-3.4)	29.7-3.5 (3.6-3.5)	29.6-3.3 (3.4-3.3)
**Space group**	*I* 4 2 2					
**Unit cell (Å)**	127.8 127.8 200.9 90 90 90	128.0 128.0 202.1 90 90 90	127.3 127.3 202.8 90 90 90	127.9 127.9 201.9 90 90 90	126.9 126.9 201.8 90 90 90	127.1 127.1 200.6 90 90 90
**Total reflections**	185745 (18472)	613930 (60014)	985401 (96603)	1056926 (102926)	427936 (43203)	509789 (51498)
Unique reflections	12883 (1260)	23425 (1270)	11812 (449)	11874 (446)	10747 (728)	12718 (472)
Multiplicity	14.4 (14.7)	26.2 (26.2)	83.4 (83.3)	89.0 (89.7)	39.8 (40.8)	40.1 (41.8)
Completeness (%)	99.66 (99.92)	94.81 (55.41)	91.84 (38.71)	92.05 (38.85)	96.59 (68.68)	92.63 (38.31)
Mean I/sigma(I)	24.82 (3.50)	24.01 (1.44)	14.08 (1.13)	17.87 (1.18)	20.78 (1.43)	19.61 (1.54)
$\boldsymbol{R}_{\mathbf{merge}}$	0.0670 (0.8552)	0.0894 (2.63)	0.8998 (20.46)	0.5776 (8.902)	0.183 (3.537)	0.252 (3.206)
$\boldsymbol{R}_{\mathbf{pim}}$	0.0186 (0.2298)	0.0181 (0.5217)	0.0988 (2.247)	0.0612 (0.9409)	0.0296 (0.5582)	0.0403 (0.4995)
**CC1/2**	1 (0.945)	0.999 (0.883)	0.999 (0.701)	0.999 (0.826)	1 (0.56)	1 (0.839)
$\boldsymbol{R}_{\mathbf{work}}$	0.255 (0.300)	0.250 (0.329)	0.259 (0.324)	0.249 (0.268)	0.291 (0.462)	0.262 (0.344)
$\boldsymbol{R}_{\mathbf{free}}$	0.272	0.264	0.280	0.261	0.297	0.281

	(0.260)	(0.331)	(0.406)	(0.361)	(0.490)	(0.369)
**Non-hydrogen atoms No.**	2376	2304	2298	2339	2301	2260
**Macromolecules**	1842	1816	1813	1856	1815	1778
**ligands**	517	470	472	472	472	472
**solvent**	17	18	13	11	14	10
**Protein residues**	225	222	222	228	223	218
**RMS (bonds)**	0.012	0.009	0.012	0.010	0.004	0.010
**RMS (angles)**	1.45	1.02	1.46	1.09	0.64	1.08
**Ramachandran favoured (%)**	96.38	98.62	99.08	98.23	98.63	98.13
**Ramachandran allowed (%)**	3.62	1.38	0.92	1.77	1.37	1.87
**Ramachandran outliers (%)**	0.00	0.00	0.00	0.00	0.00	0.00
**Average B-factor**	111.53	94.21	69.11	67.60	155.29	91.09
**Macromolecules**	105.92	87.72	64.71	59.35	149.17	85.88
**ligands**	132.49	120.09	86.53	100.70	179.65	111.39

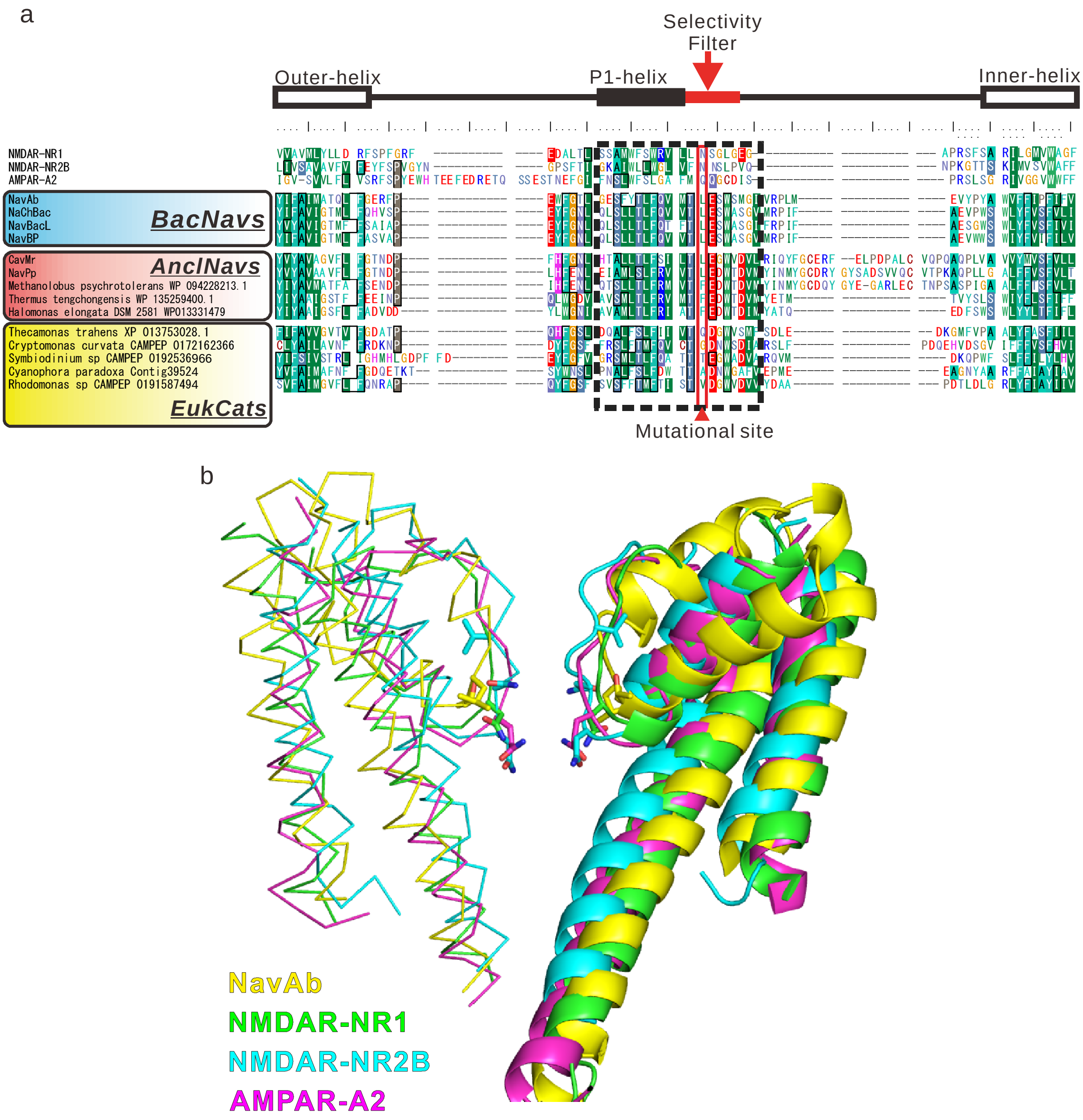

Supplementary Figure 1.The alignment of primary sequence and 3D structure of NavAb pore domain with other tetrameric ion channels.

a) Alignment of the amino-acid sequences of the P1 helix and the selectivity filter of NMDAR NR1 and NR2b subunit (pdb code; 4tlm), AMPAR A2 subunit (pdb code; 5wek), BacNavs, AnclNavs, and EukCats. The superimposed region is indicated by a dashed black line. The red arrow indicates the region of the selectivity filter. The red arrowhead indicates the mutational site in this study. Accession numbers including "CAMPEP" derive from the MMETSP database.

b) The pore domain of NavAb N49K mutant (pdb code; 5yuc) superimposed on that of NMDAR NR1 and NR2b subunit (pdb code; 4tlm), and AMPAR A2 subunit (pdb code;

5wek), respectively. Pore domains were aligned with the P1 helix, the selectivity filter and the P2 helix (NavAb) and the pore helix and the selectivity filter (NMDAR and AMPAR). The NavAb and the NMDAR NR1 and NR2b and AMPA A2 subunits are coloured yellow, green, cyan, and magenta, respectively.

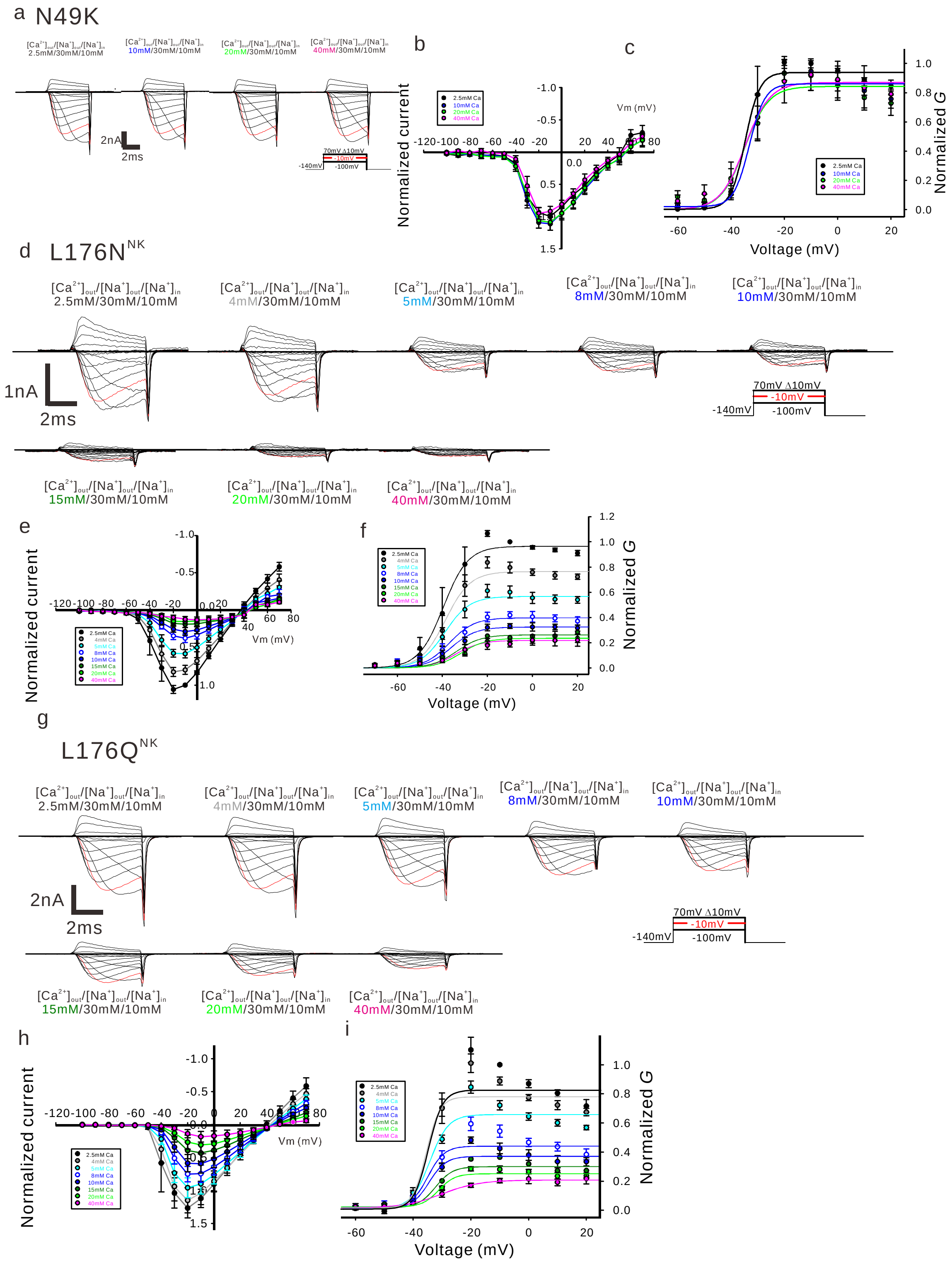

Supplementary Figure 2. The current trace, peak current, and tail current of NavAb N49K, L176N^{NK}, and L176Q^{NK} mutants

a). Representative current traces to obtain the peak current and the deactivation tail current to evaluate the current-voltage relationship and the voltage-dependent activation of NavAb N49K mutant. b). Current-voltage relationships of NavAb N49K mutant measured under various extracellular Ca^{2+} concentrations (*n* = 6). All values were normalized by the peak current generated by -10mV stimulation pulse under 2.5 mM extracellular Ca^{2+} condition. c). Normalized *G* curve of NavAb N49K mutant derived from the tail currents in various extracellular Ca^{2+} concentrations (*n* = 4). All values were normalized by the tail current generated by -10mV stimulation pulse under 2.5 mM extracellular Ca^{2+} condition. d) Representative current traces to obtain the peak current and the deactivation tail current to evaluate the current-voltage relationship and the voltage-dependent activation of NavAb $L176N^{NK}$ mutant. e). Current-voltage relationships of NavAb $L176N^{NK}$ mutant measured under various extracellular Ca^{2+} concentrations (*n* = 5). All values were normalized by the peak current generated by -10mV stimulation pulse under 2.5 mM extracellular Ca^{2+} condition. f). Normalized *G* curve of NavAb $L176N^{NK}$ mutant derived from the tail currents in various extracellular Ca^{2+} concentrations (*n* = 5). All values were normalized by the tail current generated by -10mV stimulation pulse under 2.5 mM extracellular Ca^{2+} condition. g) Representative current traces to obtain the peak current and the deactivation tail current to evaluate the current-voltage relationship and the voltage-dependent activation of NavAb $L176Q^{NK}$ mutant. h). Current-voltage relationships of NavAb $L176Q^{NK}$ mutant measured under various extracellular Ca^{2+} concentrations (*n* = 3). All values were normalized by the peak current generated by -10mV stimulation pulse under 2.5 mM extracellular Ca^{2+} condition. i). Normalized *G* curve of NavAb $L176Q^{NK}$ mutant derived from the tail currents in various extracellular Ca^{2+} concentrations (*n* = 5). All values were normalized by the tail current generated by -10mV stimulation pulse under 2.5 mM extracellular Ca^{2+} condition. The data were obtained from biologically independent cells. Symbols and error bars indicate the average and the standard error of means of normalized current, respectively.

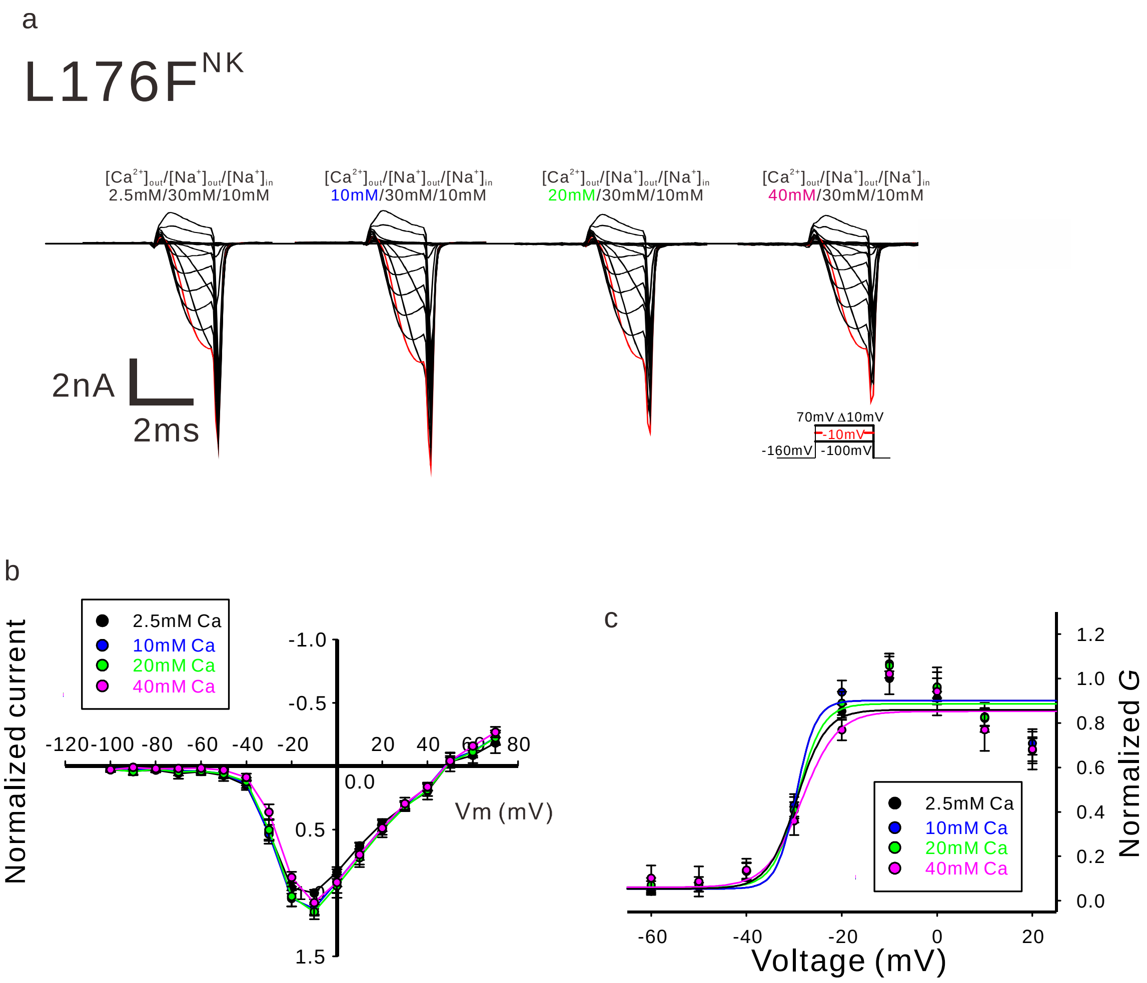

Supplementary Figure 3. The current trace, peak current, and tail current of NavAb L176F^{NK} mutant

a). Representative current traces of NavAb L176F^{NK} mutant generated by -10 mV stimulation pulses in various extracellular Ca^{2+} concentration solutions. b). Current-voltage relationships of NavAb L176F^{NK} mutant measured under various extracellular Ca^{2+} concentrations (n = 5). All values were normalized by the peak current generated by -10mV stimulation pulse under 2.5 mM extracellular Ca^{2+} condition. c). Normalized G curve of NavAb L176F^{NK} mutant derived from the tail currents in various extracellular Ca^{2+} concentrations (n = 6). All values were normalized by the tail current generated by -10mV stimulation pulse under 2.5 mM extracellular Ca^{2+} condition. The data were obtained from biologically independent cells. Symbols and error bars indicate the average and the standard error of means of normalized current, respectively.

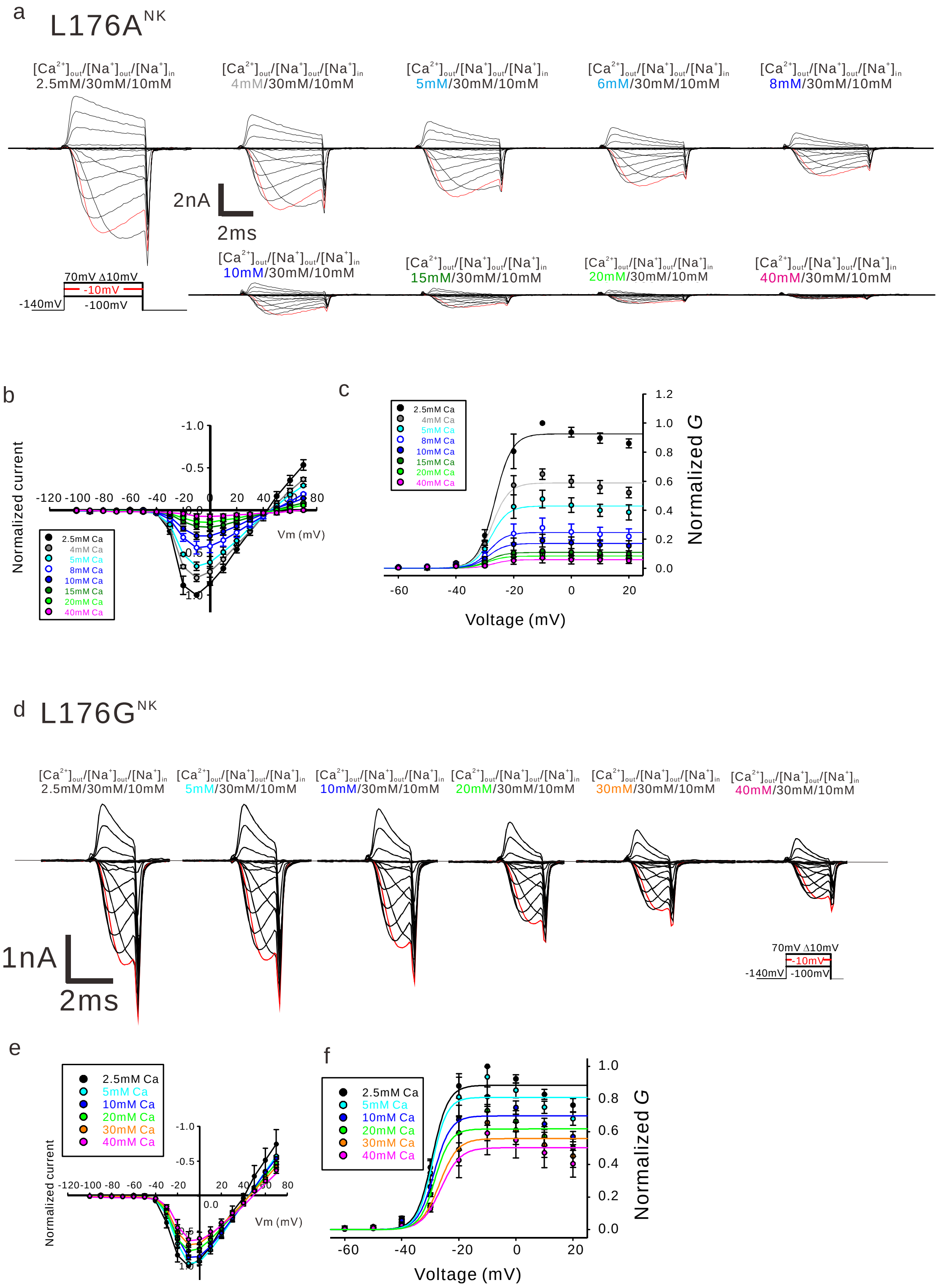

Supplementary Figure 4. The current trace, peak current, and tail current of NavAb L176A^{NK} and L176G^{NK} mutants

a). Representative current traces to obtain the peak current and the deactivation tail current to evaluate the current-voltage relationship and the voltage-dependent activation

of NavAb L176A^{NK} mutant. b). Current-voltage relationships of NavAb L176A^{NK} mutant measured under various extracellular Ca^{2+} concentrations (n = 4). All values were normalized by the peak current generated by -10mV stimulation pulse under 2.5 mM extracellular Ca^{2+} condition. c). Normalized G curve of NavAb L176A^{NK} mutant derived from the tail currents in various extracellular Ca^{2+} concentrations (n = 4). All values were normalized by the tail current generated by -10mV stimulation pulse under 2.5 mM extracellular Ca^{2+} condition. d). Representative current traces to obtain the peak current and the deactivation tail current to evaluate the current-voltage relationship and the voltage-dependent activation of NavAb L176G^{NK} mutant. e). Current-voltage relationships of NavAb L176G^{NK} mutant measured under various extracellular Ca^{2+} concentrations (n = 4). All values were normalized by the peak current generated by -10mV stimulation pulse under 2.5 mM extracellular Ca^{2+} condition. f). Normalized G curve of NavAb L176G^{NK} mutant derived from the tail currents in various extracellular Ca^{2+} concentrations (n = 4). All values were normalized by the tail current generated by -10mV stimulation pulse under 2.5 mM extracellular Ca^{2+} condition. The data were obtained from biologically independent cells. Symbols and error bars indicate the average and the standard error of means of normalized current, respectively.

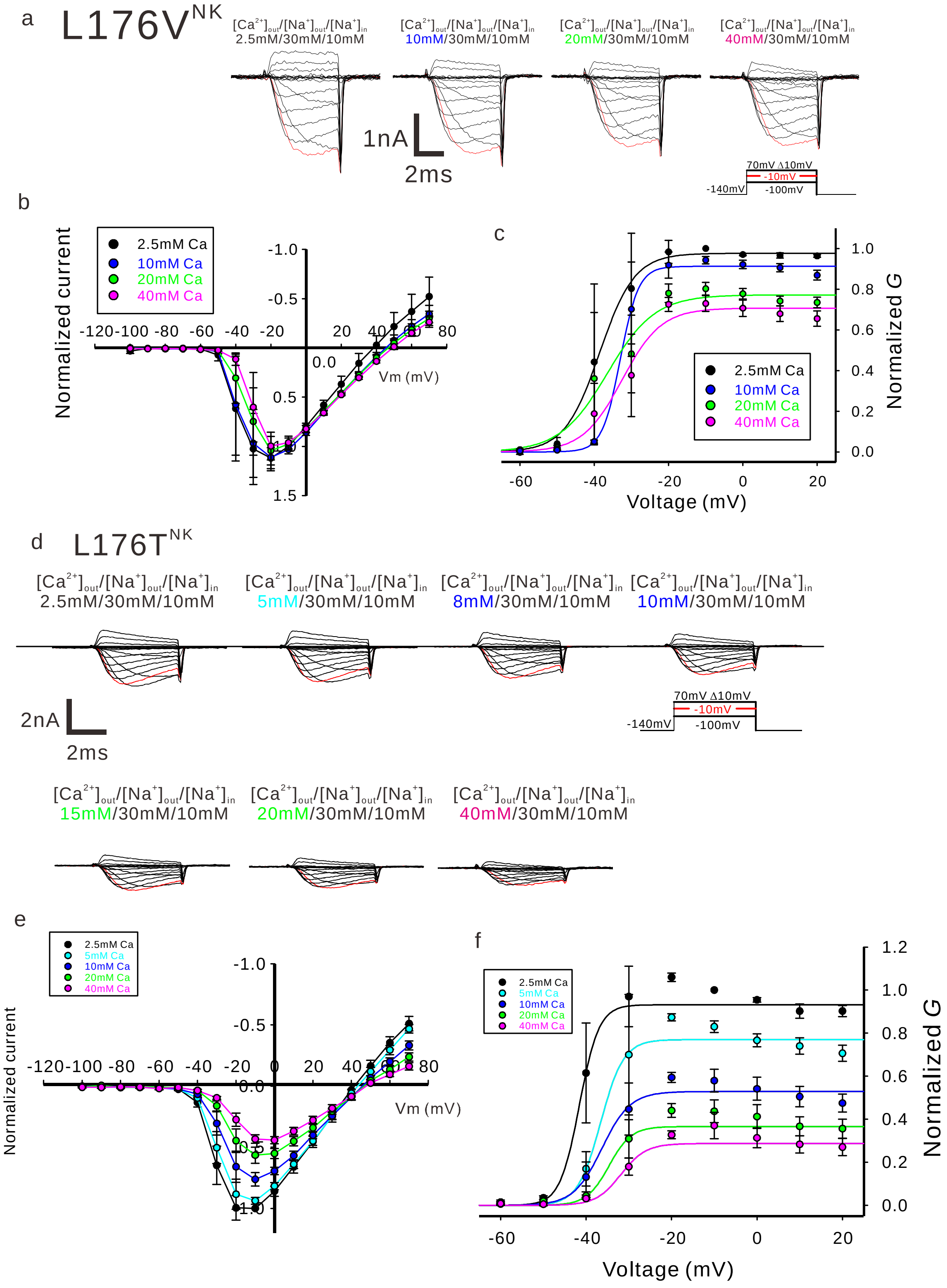

Supplementary Figure 5. The current trace, peak current, and tail current of NavAb L176V^{NK} and L176T^{NK} mutants

a). Representative current traces of NavAb L176V^{NK} mutant generated by -10 mV stimulation pulses in various extracellular Ca^{2+} concentration solutions. b). Current-voltage relationships of NavAb L176V^{NK} mutant measured under various extracellular Ca^{2+} concentrations (n = 3). All values were normalized by the peak current generated by -10mV stimulation pulse under 2.5 mM extracellular Ca^{2+} condition. c). Normalized *G* curve of NavAb L176V^{NK} mutant derived from the tail currents in various extracellular Ca^{2+} concentrations (n = 3). All values were normalized by the tail current generated by -10mV stimulation pulse under 2.5 mM extracellular Ca^{2+} condition. d) Representative current traces to obtain the peak current and the deactivation tail current to evaluate the current-voltage relationship and the voltage-dependent activation of NavAb L176T^{NK} mutant. e). Current-voltage relationships of NavAb L176T^{NK} mutant measured under various extracellular Ca^{2+} concentrations (n = 4). All values were normalized by the peak current generated by -10mV stimulation pulse under 2.5 mM extracellular Ca^{2+} condition. f). Normalized *G* curve of NavAb L176T^{NK} mutant derived from the tail currents in various extracellular Ca^{2+} concentrations (n = 4). All values were normalized by the tail current generated by -10mV stimulation pulse under 2.5 mM extracellular Ca^{2+} condition. The data were obtained from biologically independent cells. Symbols and error bars indicate the average and the standard error of means of normalized current, respectively.

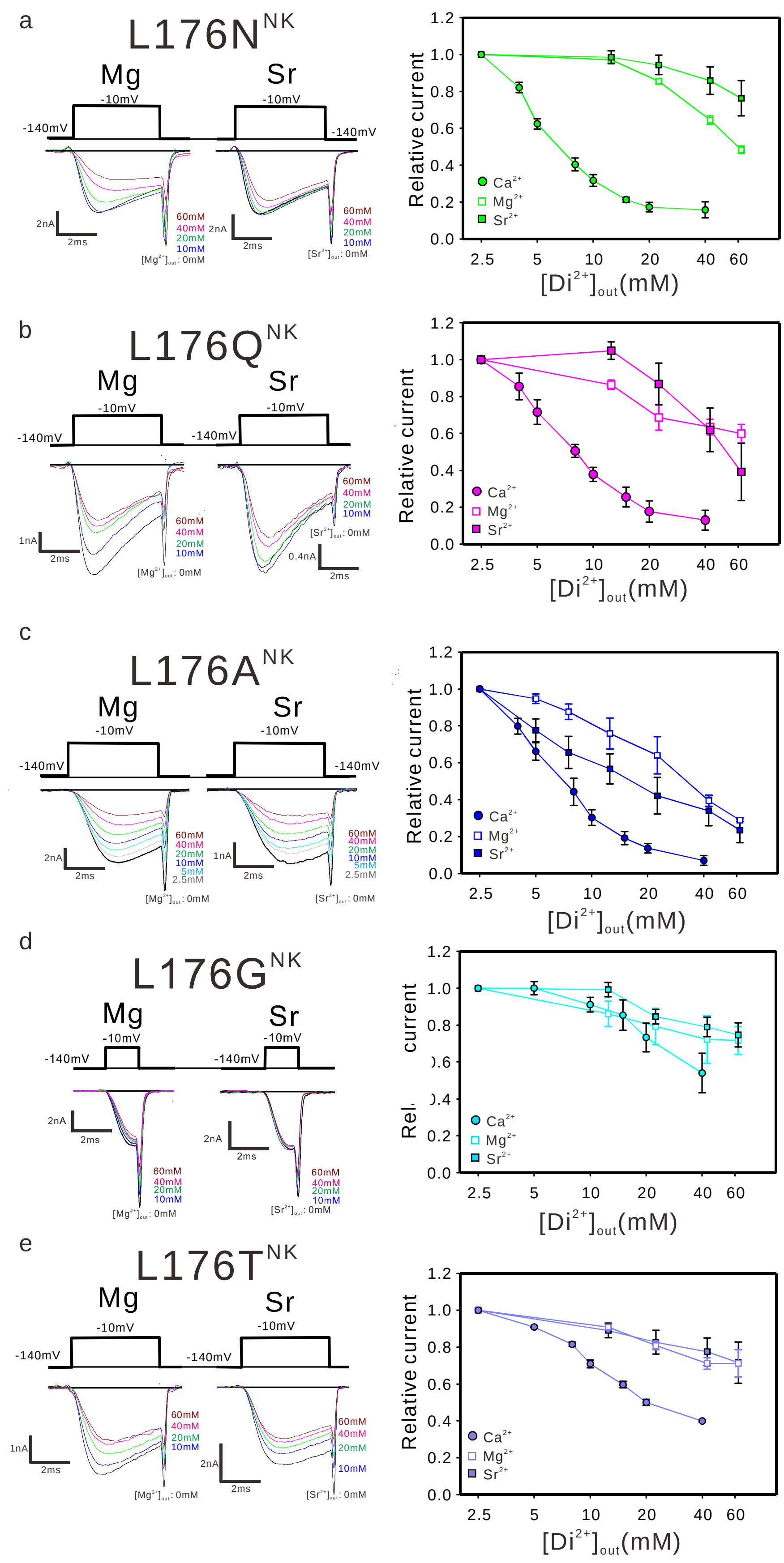
a
L176N^NK
Mg
Sr
-10mV
-140mV
Relative current
Ca2+
Mg2+
Sr2+
[Di2+]out(mM)
b
L176Q^NK
c
L176A^NK
d
L176G^NK
e
L176T^NK

Supplementary Figure 6. The blocking effect of magnesium and strontium ion

a-e). Left: Representative current traces of NavAb L176N^{NK}, L176Q^{NK}, L176A^{NK}, L176G^{NK}, and L176T^{NK} mutant generated by -10 mV stimulation pulses in various extracellular magnesium and strontium concentration solutions, respectively. Right: Relative current generated by -10 mV stimulation pulses in various extracellular Ca^{2+}, Mg^{2+}, and Sr^{2+} concentrations. All values were normalized by the tail current generated by -10mV stimulation pulse under 2.5 mM extracellular Ca^{2+} condition (n = 3). The data were obtained from biologically independent cells. Symbols and error bars indicate the average and the standard error of means of relative current normalized to the current at 2.5 mM divalent cation ion concentration conditions, respectively.

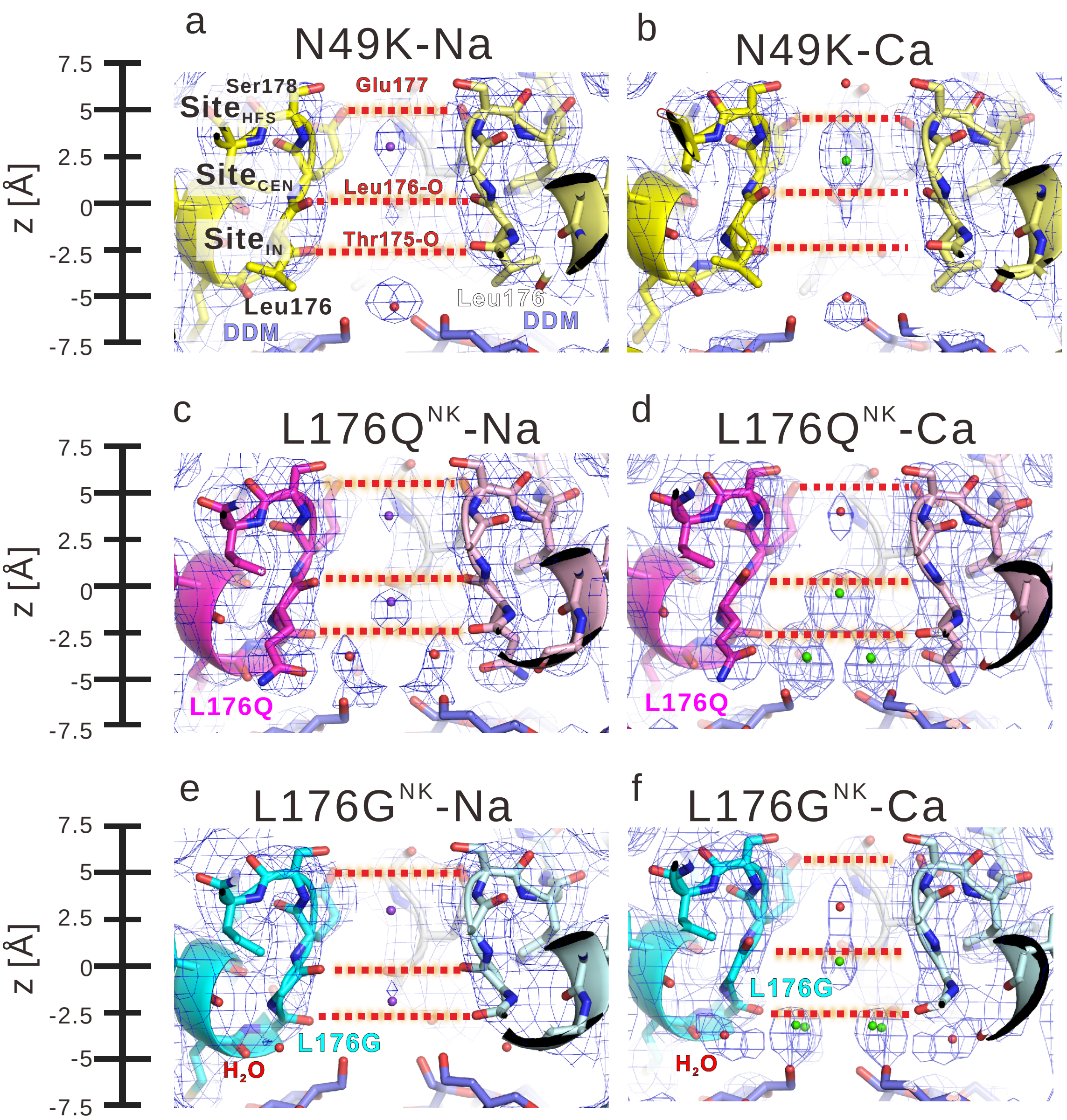

Supplementary Figure 7. The electron density map of the NavAb N49K, L176Q^{NK,} and L176G^{NK} mutants.

(a-f) Horizontal view of the electron densities of the ion pathway of NavAb N49K, L176Q^{NK,} and L176G^{NK} mutants in the non-calcium and calcium conditions. The upside is the extracellular side. Red dashed lines between Site$_{HFS}$, Site$_{CEN}$, and Site$_{IN}$ indicate the interaction site of the selectivity filter, high-field-strength site, centre site, and inner site, respectively. Blue mesh indicates the $2F_O - F_C$ electron density map contoured at 1σ.

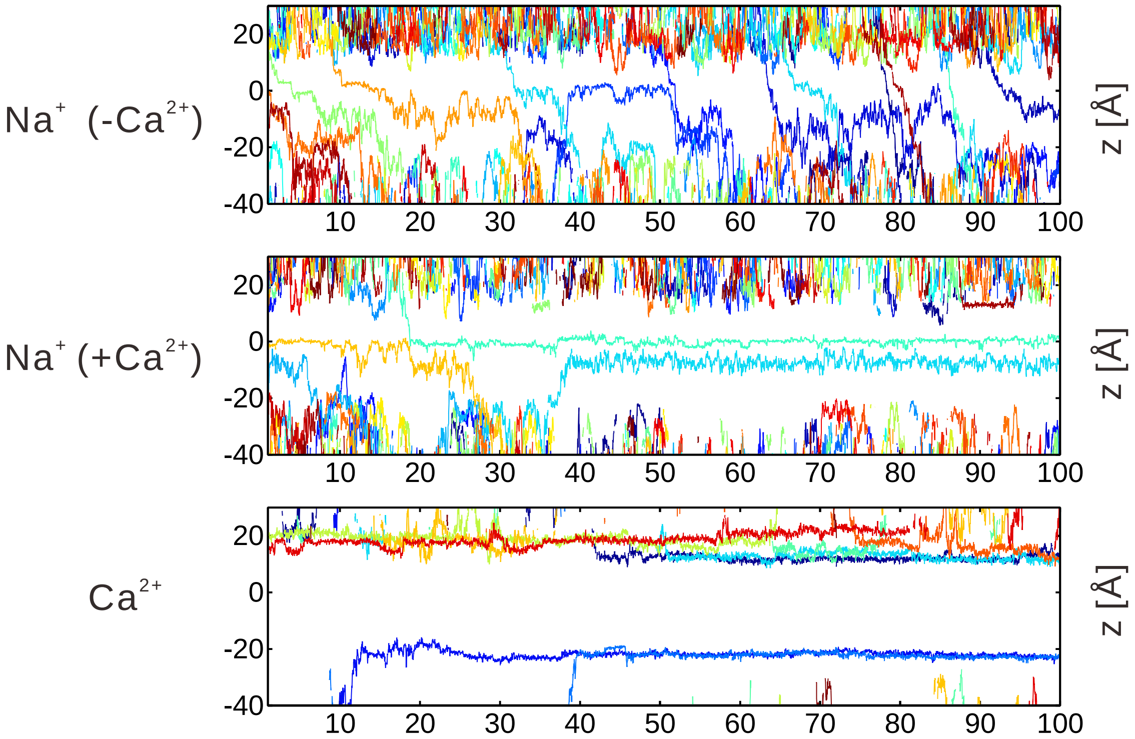

Supplementary Figure 8. The ionic trajectories of the NavAb wild-type channel calculated in MD simulations without the electronic continuum correction.

The trajectories of repeated ion permeation across the pore are shown along the z-axis as a function of time (t). Z-axis values correspond to that in Fig. 4a.

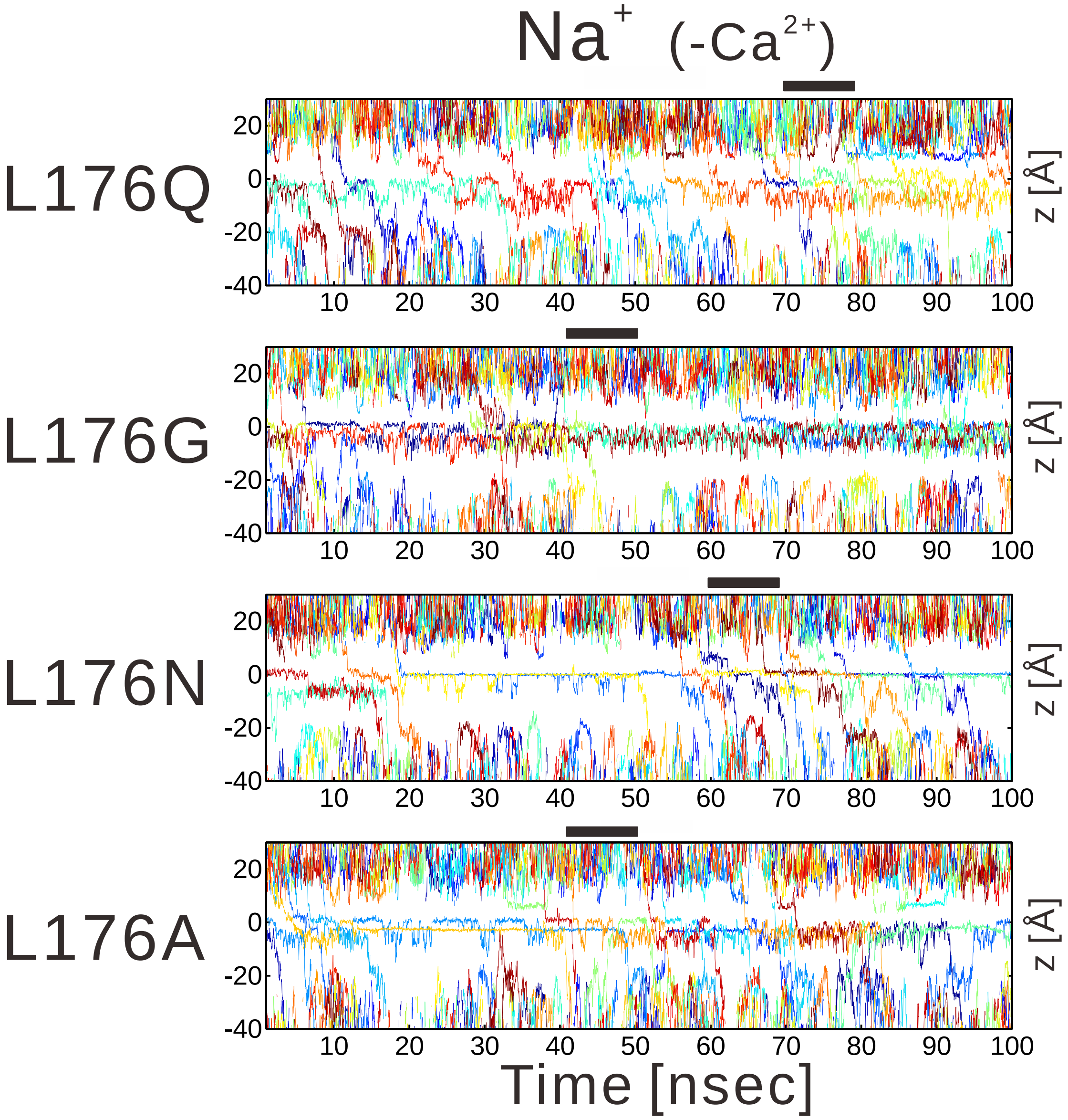

Supplementary Figure 9. The sodium ion trajectories of NavAb L176Q, L176G, L176N, and L176A mutants without calcium ions calculated in MD simulations. The trajectories of repeated ion permeation across the pore are shown along the z-axis as a function of time (t). The black bars indicate the time period used for evaluating water concentration in Supplementary Fig. 13. Z-axis values correspond to that in Fig. 4a.

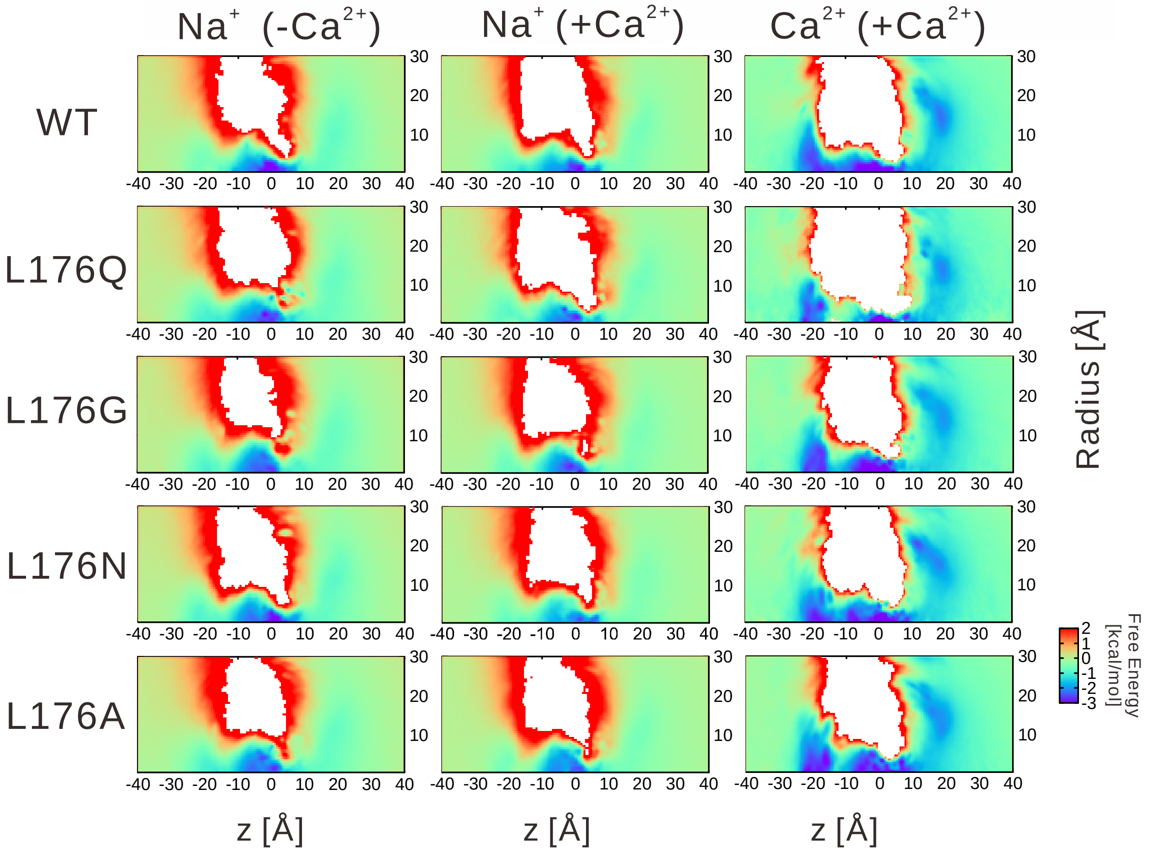

Supplementary Figure 10. Two-dimensional free energy landscape of the ions in each mutant.

Free energy was plotted with radius on the vertical axis and z-axis on the horizontal axis. The columns of Na^{+} ($-Ca^{2+}$) and Na^{+} ($+Ca^{2+}$) indicate the 2D free energy of sodium ions in total simulation times of each mutant calcium-free and calcium condition, respectively. The columns of Ca^{2+} ($+Ca^{2+}$) indicate the 2D free energy of calcium ions in the total simulation times of each mutant calcium condition. The values of the radius and z-axis correspond to those shown in Fig. 4-6. The colour scale indicates the free energy values in the lower right.

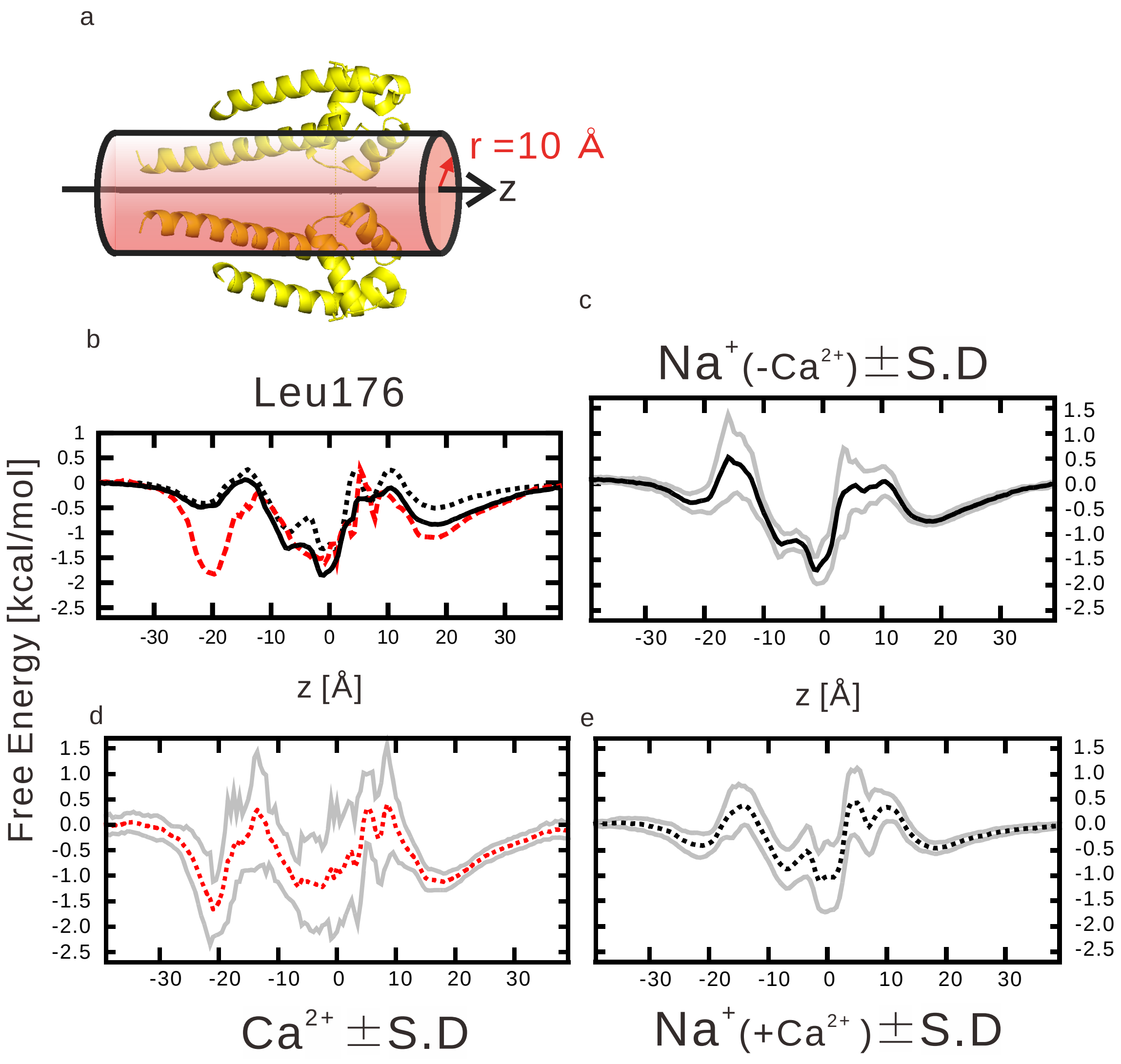

Supplementary Figure 11. The one-dimensional landscape of the free energy in the whole system with the standard deviation.

a) Schematic diagram showing the region used to calculate one-dimensional free energy. b) The one-dimensional landscape of the ionic free energy of NavAb wild-type channel among periodic repeats. Black and dashed-black lines indicated the free energy of sodium ions in calcium-free conditions and calcium conditions, respectively. The dashed-red line indicates the free energy of calcium ions in calcium conditions. c-e) The one-dimensional landscape of the free energy in the whole system with the standard deviation. Black, dashed-black, and dashed-red lines indicated the averaged free energy of each 100-nsec simulation of sodium ions in calcium-free conditions (c) and calcium conditions (d), and calcium ions (e), respectively. Grey lines indicated the value of the averaged free energy ± stander deviation.

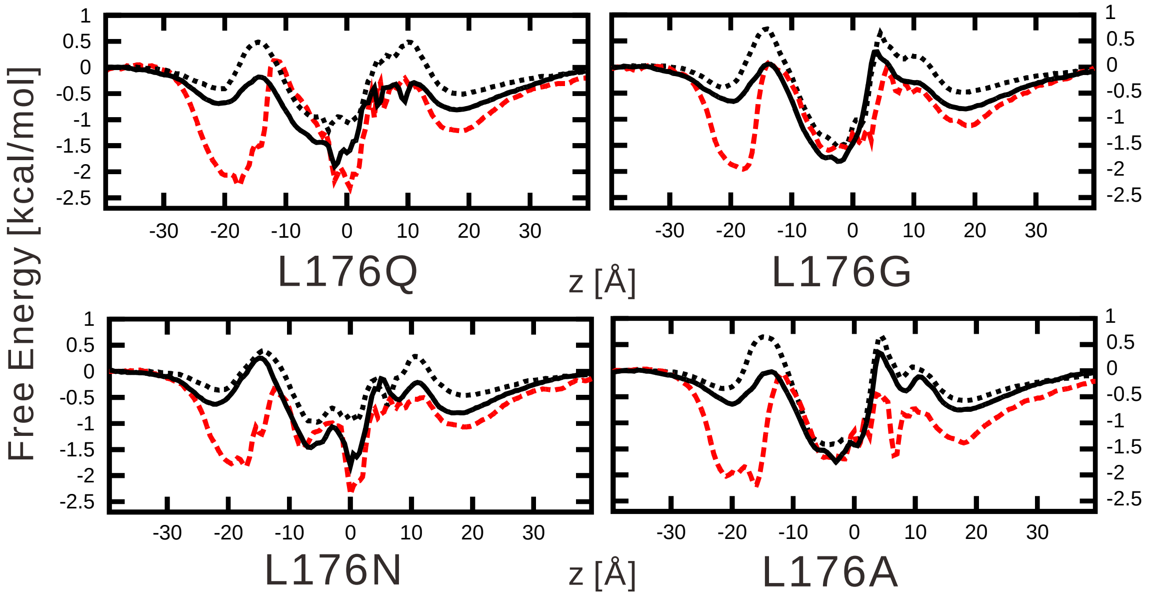

Supplementary Figure 12. The one-dimensional landscape of the ionic free energy of NavAb mutant channels among periodic repeats.

Black and dashed black lines indicated the free energy of sodium ions in calcium-free conditions and calcium conditions, respectively. The dashed-red line indicates the free energy of calcium ions in calcium conditions.

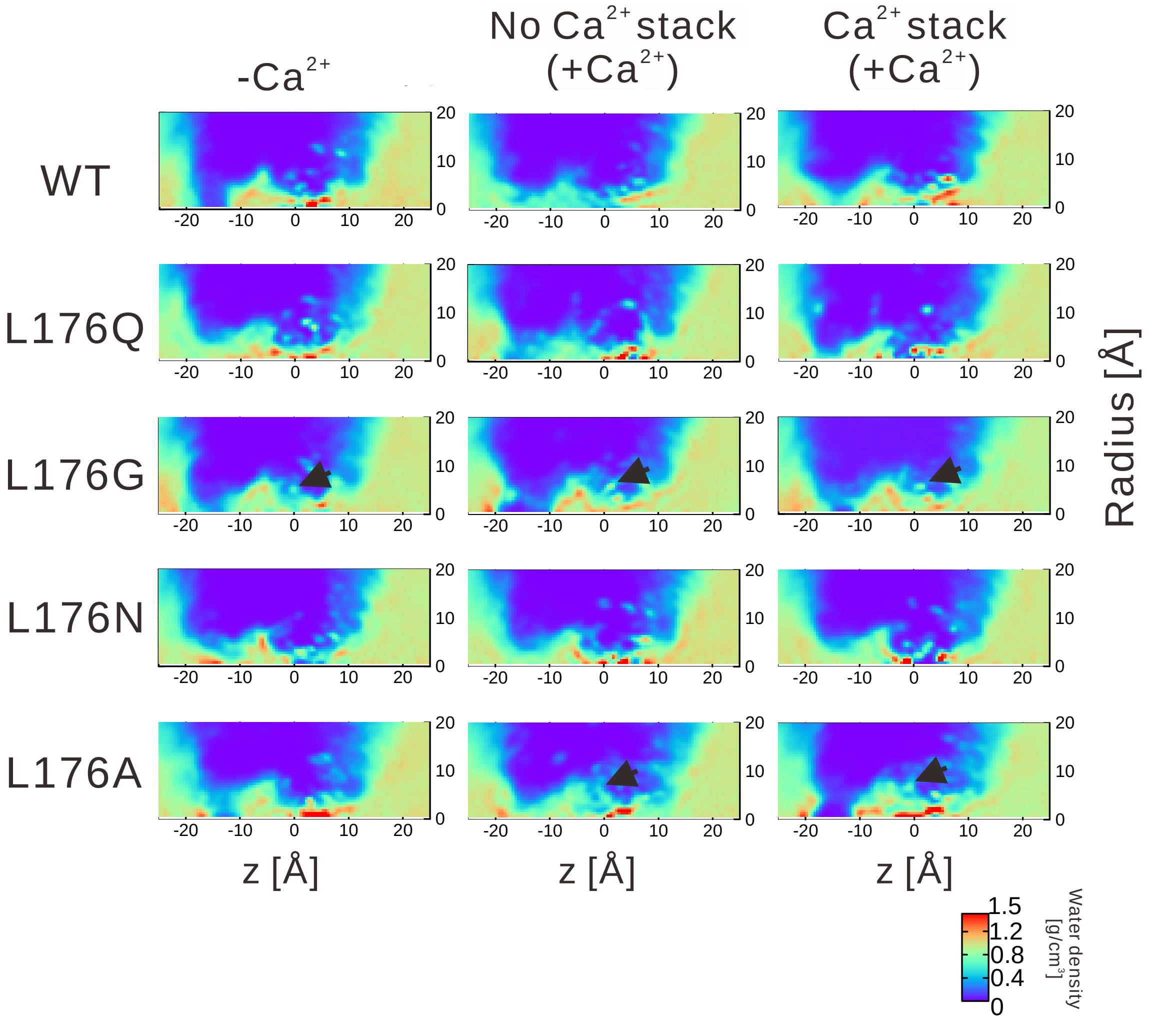

Supplementary Figure 13. The two-dimensional landscape of water density in each mutant and ionic condition.

Water density was plotted with radius on the vertical axis and z-axis on the horizontal axis. The columns of -Ca^{2+} are the water density at the period indicated by the black bar in Fig. 5b or Supplementary Fig. 9. The columns of No Ca^{2+} stack (+Ca^{2+}) and Ca^{2+} stack (+Ca^{2+}) are the water density at the period indicated by the white and black bar in Fig. 5c, respectively. The values of the radius and z-axis correspond to those shown in Fig. 4-6. The colour scale indicates the water density values in the lower right. The black arrows indicate water in the extra cavity.
