# Supplementary material for: The structural basis of divalent cation block in a tetrameric prokaryotic sodium channel
Source: Nat Commun. 2023 Jul 15;14:4236. doi: 10.1038/s41467-023-39987-0 (PMC10349818; doi:10.1038/s41467-023-39987-0)
Supplement: Supplementary file 3 — Description of Additional Supplementary Files [file 41467_2023_39987_MOESM3_ESM.pdf]

Supplementary Movie 1. The electron density map of the NavAb N49K mutant around the selectivity filter in non-calcium condition.

Horizontal view of the electron densities of the ion pathway of NavAb N49K mutants in the non-calcium conditions. The upside is the extracellular side. Blue mesh indicates the 2FO − FC electron density map contoured at 1σ.

Supplementary Movie 2. The electron density map of the NavAb N49K mutant around the selectivity filter in the calcium condition.

Horizontal view of the electron densities of the ion pathway of NavAb N49K mutants in the calcium conditions. The upside is the extracellular side. Blue mesh indicates the 2FO − FC electron density map contoured at 1σ.

Supplementary Movie 3. The electron density map of the NavAb L176QNK mutant around the selectivity filter in the non-calcium

Horizontal view of the electron densities of the ion pathway of NavAb L176QNK mutants in the non-calcium conditions. The upside is the extracellular side. Blue mesh indicates the 2FO − FC electron density map contoured at 1σ.

Supplementary Movie 4. The electron density map of the NavAb L176QNK mutant around the selectivity filter in the calcium condition.

Horizontal view of the electron densities of the ion pathway of NavAb L176QNK mutants in the calcium conditions. The upside is the extracellular side. Blue mesh indicates the 2FO − FC electron density map contoured at 1σ.

Supplementary Movie 5. The electron density map of the NavAb L176GNK mutant around the selectivity filter in the non-calcium

Horizontal view of the electron densities of the ion pathway of NavAb L176GNK mutants in the non-calcium conditions. The upside is the extracellular side. Blue mesh indicates the 2FO − FC electron density map contoured at 1σ.

Supplementary Movie 6. The electron density map of the NavAb L176GNK mutant around the selectivity filter in the calcium condition.

Horizontal view of the electron densities of the ion pathway of NavAb L176GNK mutants in the calcium conditions. The upside is the extracellular side. Blue mesh indicates the 2FO − FC electron density map contoured at 1σ.

Supplementary Movie 7. The ionic permeation process of the NavAb wild-type channel in non-equilibrium MD simulation without the electronic continuum correction under the non-calcium condition.

The 100-nsec movie of the ionic permeation process of the NavAb wild-type channel calculated in non-equilibrium MD simulation without the electronic continuum correction under the non-calcium condition. The transmembrane and pore helices of NavAb (gray cylinder) form the inner vestibule. The stick model indicates the selectivity-filter residues. The line model indicates water molecules. The purple sphere indicates a sodium ion. The green sphere indicates a calcium ion. Red spheres indicate the main chain carboxyl oxygen atoms of the 176th residue.

Supplementary Movie 8. The ionic permeation process of the NavAb wild-type channel in non-equilibrium MD simulation without the electronic continuum correction under the calcium condition.

The 100-nsec movie of the ionic permeation process of the NavAb wild-type channel calculated in non-equilibrium MD simulation without the electronic continuum correction under the calcium condition. The transmembrane and pore helices of NavAb (gray cylinder) form the inner vestibule. The stick model indicates the selectivity-filter residues. The line model indicates water molecules. The purple sphere indicates a sodium ion. The green sphere indicates a calcium ion. Red spheres indicate the main chain carbonyl oxygen atoms of the 176th residue.

Supplementary Movie 9. The ionic permeation process of the NavAb wild-type channel calculated in non-equilibrium MD simulation with the electronic continuum correction under the non-calcium condition.

The 100-nsec movie of the ionic permeation process of the NavAb wild-type channel calculated in non-equilibrium MD simulation with the electronic continuum correction under the non-calcium condition. The transmembrane and pore helices of NavAb (gray cylinder) form the inner vestibule. The stick model indicates the selectivity-filter residues. The line model indicates water molecules. The purple sphere indicates a sodium ion. The green sphere indicates a calcium ion. Red spheres indicate the main chain carbonyl oxygen atoms of the 176th residue. The black and white bars indicate the time period used for evaluating water concentration with or without stacking calcium ions in Sup. Fig13, respectively.

Supplementary Movie 10. The ionic permeation process of the NavAb wild-type channel calculated in non-equilibrium MD simulation with the electronic continuum correction under the calcium condition.

The 100-nsec movie of the ionic permeation process of the NavAb wild-type channel calculated in non-equilibrium MD simulation with the electronic continuum correction under the calcium condition. The transmembrane and pore helices of NavAb (gray cylinder) form the inner vestibule. The stick model indicates the selectivity-filter residues. The line model indicates water molecules. The purple sphere indicates a sodium ion. The green sphere indicates a calcium ion. Red spheres indicate the main chain carbonyl oxygen atoms of the 176th residue. The black and white bars indicate the time period used for evaluating water concentration with or without stacking calcium ions in Sup. Fig13, respectively.

Supplementary Movie 11. The ionic permeation process of the NavAb L176Q channel calculated in non-equilibrium MD simulation with the electronic continuum correction under the non-calcium condition.

The 100-nsec movie of the ionic permeation process of the NavAb L176Q channel calculated in non-equilibrium MD simulation with the electronic continuum correction under the non-calcium condition. The transmembrane and pore helices of NavAb (gray cylinder) form the inner vestibule. The stick model indicates the selectivity-filter residues. The line model indicates water molecules. The purple sphere indicates a sodium ion. The green sphere indicates a calcium ion. Red spheres indicate the main chain carbonyl oxygen atoms of the 176th residue. The black and white bars indicate the time period used for evaluating water concentration with or without stacking calcium ions in Sup. Fig13, respectively.

Supplementary Movie 12. The ionic permeation process of the NavAb L176Q channel calculated in non-equilibrium MD simulation with the electronic continuum correction under the calcium condition.

The 100-nsec movie of the ionic permeation process of the NavAb L176Q channel calculated in non-equilibrium MD simulation with the electronic continuum correction under the calcium condition. The transmembrane and pore helices of NavAb (gray cylinder) form the inner vestibule. The stick model indicates the selectivity-filter residues. The line model indicates water molecules. The purple sphere indicates a sodium ion. The green sphere indicates a calcium ion. Red spheres indicate the main chain carbonyl oxygen atoms of the 176th residue. The black and white bars indicate the time period used for evaluating water concentration with or without stacking calcium ions in Sup. Fig13, respectively.

Supplementary Movie 13. The ionic permeation process of the NavAb L176G channel calculated in non-equilibrium MD simulation with the electronic continuum correction under the non-calcium condition.

The 100-nsec movie of the ionic permeation process of the NavAb L176G channel calculated in non-equilibrium MD simulation with the electronic continuum correction under the non-calcium condition. The transmembrane and pore helices of NavAb (gray cylinder) form the inner vestibule. The stick model indicates the selectivity-filter residues. The line model indicates water molecules. The purple sphere indicates a sodium ion. The green sphere indicates a calcium ion. Red spheres indicate the main chain carbonyl oxygen atoms of the 176th residue. The black and white bars indicate the time period used for evaluating water concentration with or without stacking calcium ions in Sup. Fig13, respectively.

Supplementary Movie 14. The ionic permeation process of the NavAb L176G channel calculated in non-equilibrium MD simulation with the electronic continuum correction under the calcium condition.
The 100-nsec movie of the ionic permeation process of the NavAb L176G channel calculated in non-equilibrium MD simulation with the electronic continuum correction under the calcium condition. The transmembrane and pore helices of NavAb (gray cylinder) form the inner vestibule. The stick model indicates the selectivity-filter residues. The line model indicates water molecules. The purple sphere indicates a sodium ion. The green sphere indicates a calcium ion. Red spheres indicate the main chain carbonyl oxygen atoms of the 176th residue. The black and white bars indicate the time period used for evaluating water concentration with or without stacking calcium ions in Sup. Fig13, respectively.

Supplementary Movie 15. The ionic permeation process of the NavAb L176N channel calculated in non-equilibrium MD simulation with the electronic continuum correction under the non-calcium condition.
The 100-nsec movie of the ionic permeation process of the NavAb L176N channel calculated in non-equilibrium MD simulation with the electronic continuum correction under the non-calcium condition. The transmembrane and pore helices of NavAb (gray cylinder) form the inner vestibule. The stick model indicates the selectivity-filter residues. The line model indicates water molecules. The purple sphere indicates a sodium ion. The green sphere indicates a calcium ion. Red spheres indicate the main chain carbonyl oxygen atoms of the 176th residue. The black and white bars indicate the time period used for evaluating water concentration with or without stacking calcium ions in Sup. Fig13, respectively.

Supplementary Movie 16. The ionic permeation process of the NavAb L176N channel calculated in non-equilibrium MD simulation with the electronic continuum correction under the calcium condition.

The 100-nsec movie of the ionic permeation process of the NavAb L176N channel calculated in non-equilibrium MD simulation with the electronic continuum correction under the calcium condition. The transmembrane and pore helices of NavAb (gray cylinder) form the inner vestibule. The stick model indicates the selectivity-filter residues. The line model indicates water molecules. The purple sphere indicates a sodium ion. The green sphere indicates a calcium ion. Red spheres indicate the main chain carbonyl oxygen atoms of the 176th residue. The black and white bars indicate the time period used for evaluating water concentration with or without stacking calcium ions in Sup. Fig13, respectively.

Supplementary Movie 17. The ionic permeation process of the NavAb L176A channel calculated in non-equilibrium MD simulation with the electronic continuum correction under the non-calcium condition.

The 100-nsec movie of the ionic permeation process of the NavAb L176A channel calculated in non-equilibrium MD simulation with the electronic continuum correction under the non-calcium condition. The transmembrane and pore helices of NavAb (gray cylinder) form the inner vestibule. The stick model indicates the selectivity-filter residues. The line model indicates water molecules. The purple sphere indicates a sodium ion. The green sphere indicates a calcium ion. Red spheres indicate the main chain carbonyl oxygen atoms of the 176th residue. The black and white bars indicate the time period used for evaluating water concentration with or without stacking calcium ions in Sup. Fig13, respectively.

Supplementary Movie 18. The ionic permeation process of the NavAb L176A channel calculated in non-equilibrium MD simulation with the electronic continuum correction under the calcium condition.

The 100-nsec movie of the ionic permeation process of the NavAb L176A channel calculated in non-equilibrium MD simulation with the electronic continuum correction under the calcium condition. The transmembrane and pore helices of NavAb (gray cylinder) form the inner vestibule. The stick model indicates the selectivity-filter residues. The line model indicates water molecules. The purple sphere indicates a sodium ion. The green sphere indicates a calcium ion. Red spheres indicate the main chain carbonyl oxygen atoms of the 176th residue. The black and white bars indicate the time period used for evaluating water concentration with or without stacking calcium ions in Sup. Fig13, respectively.
