# Supplementary material for: The structural basis of divalent cation block in a tetrameric prokaryotic sodium channel
Source: Nat Commun. 2023 Jul 15;14:4236. doi: 10.1038/s41467-023-39987-0 (PMC10349818; doi:10.1038/s41467-023-39987-0)
Supplement: Supplementary file 22 — Reporting Summary [file 41467_2023_39987_MOESM22_ESM.pdf]

nature portfolio

Corresponding author(s): Katsumasa Irie

Last updated by author(s): Jun 19, 2023

Reporting Summary

Nature Portfolio wishes to improve the reproducibility of the work that we publish. This form provides structure for consistency and transparency in reporting. For further information on Nature Portfolio policies, see our Editorial Policies and the Editorial Policy Checklist.

Statistics

For all statistical analyses, confirm that the following items are present in the figure legend, table legend, main text, or Methods section.

n/a	Confirmed	
☐	☒	The exact sample size (*n*) for each experimental group/condition, given as a discrete number and unit of measurement
☐	☒	A statement on whether measurements were taken from distinct samples or whether the same sample was measured repeatedly
☒	☐	The statistical test(s) used AND whether they are one- or two-sided<br>*Only common tests should be described solely by name; describe more complex techniques in the Methods section.*
☒	☐	A description of all covariates tested
☐	☒	A description of any assumptions or corrections, such as tests of normality and adjustment for multiple comparisons
☐	☒	A full description of the statistical parameters including central tendency (e.g. means) or other basic estimates (e.g. regression coefficient) AND variation (e.g. standard deviation) or associated estimates of uncertainty (e.g. confidence intervals)
☒	☐	For null hypothesis testing, the test statistic (e.g. *F*, *t*, *r*) with confidence intervals, effect sizes, degrees of freedom and *P* value noted<br>*Give P values as exact values whenever suitable.*
☒	☐	For Bayesian analysis, information on the choice of priors and Markov chain Monte Carlo settings
☒	☐	For hierarchical and complex designs, identification of the appropriate level for tests and full reporting of outcomes
☒	☐	Estimates of effect sizes (e.g. Cohen's *d*, Pearson's *r*), indicating how they were calculated

Our web collection on statistics for biologists contains articles on many of the points above.

Software and code

Policy information about availability of computer code

Data collection	Patch master v2x73.(electrophysiology), NAMD2 (molecular dynamics simulation), KAMO system (X-ray diffraction collection), xds (BUILT=20220110)(X-ray diffraction collection)
Data analysis	SigmaPlot14, Gnuplot5.4.2, Pymol2.5.2,CCP4(v.7.0.078), Phenix 1.18, COOT0.9.2 IGOR 6.37

For manuscripts utilizing custom algorithms or software that are central to the research but not yet described in published literature, software must be made available to editors and reviewers. We strongly encourage code deposition in a community repository (e.g. GitHub). See the Nature Portfolio guidelines for submitting code & software for further information.

Data

Policy information about availability of data

All manuscripts must include a data availability statement. This statement should provide the following information, where applicable:

- Accession codes, unique identifiers, or web links for publicly available datasets
- A description of any restrictions on data availability
- For clinical datasets or third party data, please ensure that the statement adheres to our policy

Data Availability
The structural data generated in this study have been deposited in the Protein Data Bank under accession codes 8H9O[https://doi.org/10.2210/pdb8H9O/pdb]: Crystal structure of voltage-gated sodium channel NavAb N49K mutant in sodium ion condition, 8H9W[https://doi.org/10.2210/pdb8H9W/pdb]: Crystal structure of

voltage-gated sodium channel NavAb N49K mutant in calcium ion condition, 8H9X[https://doi.org/10.2210/pdb8H9X/pdb]: Crystal structure of voltage-gated sodium channel NavAb N49K/L176Q mutant in sodium ion condition, 8H9Y[https://doi.org/10.2210/pdb8H9Y/pdb]: Crystal structure of voltage-gated sodium channel NavAb N49K/L176Q mutant in calcium ion condition, 8HA1[https://doi.org/10.2210/pdb8HA1/pdb]: Crystal structure of voltage-gated sodium channel NavAb N49K/L176G mutant in sodium ion condition, 8HA2[https://doi.org/10.2210/pdb8HA1/pdb]: Crystal structure of voltage-gated sodium channel NavAb N49K/L176G mutant in calcium ion condition. The structure of NavAb N49K mutant for the initial model of molecular replacement and the molecular dynamics simulation was available in the Protein Data Bank under accession codes 5YUC. The trajectories of molecular dynamics simulation that support this study are available from the corresponding author, K.I, upon reasonable request. Because of the data amount, the entire MD trajectory data are available from the corresponding author, K.I, upon reasonable request. The data that support the findings of this study are openly available in figshare at https://doi.org/10.6084/m9.figshare.22847747, reference number 22847747.
Code Availability
The initial coordinates, input file, optimized parameter, and final coordinates of MD simulation are also given to figshare at https://doi.org/10.6084/m9.figshare.22847747, reference number 22847747.

Research involving human participants, their data, or biological material

Policy information about studies with human participants or human data. See also policy information about sex, gender (identity/presentation), and sexual orientation and race, ethnicity and racism.

Reporting on sex and gender	n/a
Reporting on race, ethnicity, or other socially relevant groupings	n/a
Population characteristics	n/a
Recruitment	n/a
Ethics oversight	n/a

Note that full information on the approval of the study protocol must also be provided in the manuscript.

Field-specific reporting

Please select the one below that is the best fit for your research. If you are not sure, read the appropriate sections before making your selection.

☒ Life sciences ☐ Behavioural & social sciences ☐ Ecological, evolutionary & environmental sciences

For a reference copy of the document with all sections, see nature.com/documents/nr-reporting-summary-flat.pdf

Life sciences study design

All studies must disclose on these points even when the disclosure is negative.

Sample size	Because the channel properties varied little in each mutant, and sufficiently converged, the sample sizes described in manuscript were used.
Data exclusions	No data was excluded.
Replication	Biochemical assays, including protein expression, crystallization were examined at least three times independently. Electrophysiological experiments were repeated more than 3 times with same cell to confirm that there was no change. Each data was taken from distinct cells. This number of samples is described in the text. We confirmed the replicated experiments were successful.
Randomization	n/a. Because animals or human research participants were not involved in this study, sample were not randomized for the experiments.
Blinding	n/a. Because animals or human research participants were not involved in this study, no blinding was used in this study.

Reporting for specific materials, systems and methods

We require information from authors about some types of materials, experimental systems and methods used in many studies. Here, indicate whether each material, system or method listed is relevant to your study. If you are not sure if a list item applies to your research, read the appropriate section before selecting a response.

Materials & experimental systems

n/a	Involved in the study
☒	☐ Antibodies
☐	☒ Eukaryotic cell lines
☒	☐ Palaeontology and archaeology
☒	☐ Animals and other organisms
☒	☐ Clinical data
☒	☐ Dual use research of concern
☒	☐ Plants

Methods

n/a	Involved in the study
☒	☐ ChIP-seq
☒	☐ Flow cytometry
☒	☐ MRI-based neuroimaging

Eukaryotic cell lines

Policy information about cell lines and Sex and Gender in Research

Cell line source(s)	SF-9 cells (ATCC catalogue number CRL-1711)
Authentication	No further authentication was performed for cell lines.
Mycoplasma contamination	Not tested.
Commonly misidentified lines (See ICLAC register)	No commonly misidentified cell lines were used.
